# Photoacoustic Resonators for Non-Invasive Blood Glucose Detection Through Photoacoustic Spectroscopy: A Systematic Review

**DOI:** 10.3390/s24216963

**Published:** 2024-10-30

**Authors:** Md Rejvi Kaysir, Thasin Mohammad Zaman, Shazzad Rassel, Jishen Wang, Dayan Ban

**Affiliations:** 1 Department of Electrical and Electronic Engineering (EEE), Khulna University of Engineering & Technology (KUET), Khulna 9203, Bangladesh; 2 Photonics Research Group, Department of Electrical and Electronic Engineering (EEE), Khulna University of Engineering & Technology (KUET), Khulna 9203, Bangladesh; 3 Department of Electrical and Computer Engineering, Tennessee State University, 3500 John A Merritt Blvd, Nashville, TN 37209, USA; 4 Department of Electrical and Computer Engineering, University of Waterloo, 200 University Ave. W, Waterloo, ON N2L 3G1, Canadadban@uwaterloo.ca (D.B.); 5 Waterloo Institute for Nanotechnology, University of Waterloo, 200 University Ave. W, Waterloo, ON N2L 3G1, Canada

**Keywords:** diabetes, non-invasive glucose detection, photoacoustic spectroscopy, photoacoustic cell/resonator, acoustic amplifier, Q-factor, frequency response

## Abstract

Diabetes mellitus is a prevalent disease with a rapidly increasing incidence projected worldwide, affecting both industrialized and developing regions. Effective diabetes management requires precise therapeutic strategies, primarily through self-monitoring of blood glucose levels to achieve tight glycemic control, thereby mitigating the risk of severe complications. In recent years, there have been significant advancements in non-invasive techniques for measuring blood glucose using photoacoustic spectroscopy (PAS), as it shows great promise for the detection of glucose using the infrared region (e.g., MIR and NIR) of light. A critical aspect of this method is the detection of the photoacoustic signal generated from blood glucose, which needs to be amplified through a photoacoustic resonator (PAR). In this work, an overview of various types of PARs used for non-invasive glucose sensing is reviewed, highlighting their operating principle, design requirements, limitations, and potential improvements needed to enhance the analysis of photoacoustic signals. The motivation behind this review is to identify and discuss main parameters crucial to the efficient design of PARs used in non-invasive glucose detection, which will be helpful for furthering the basic understanding of this technology and achieving the highly sensitive PAR required for non-invasive glucose monitoring.

## 1. Introduction

**Figure 1:**
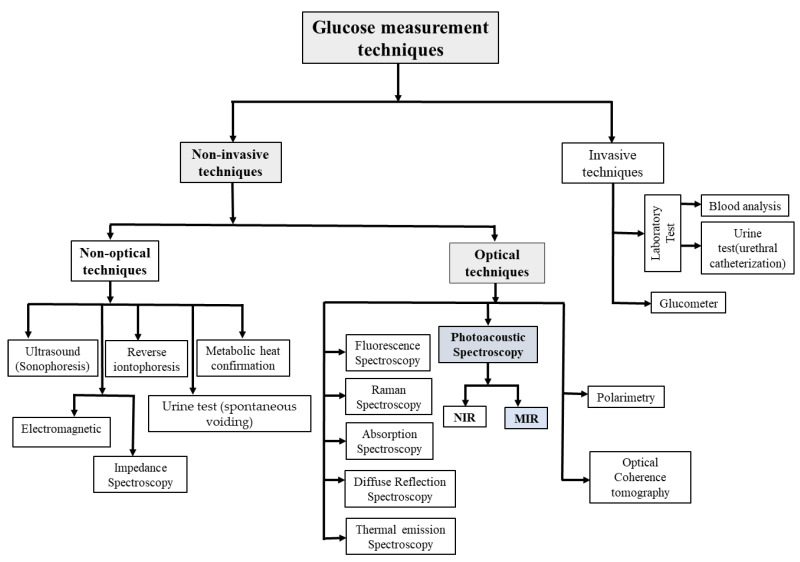
General classification of overall glucose measurement techniques covering both invasive and non-invasive methods.

Diabetes is one of the four major types of noncommunicable diseases (NCDs) leading to slow damage to the heart, blood vessels, eyes, kidneys, and nerves. The predominant form is type 2 diabetes, which typically affects adults and is characterized by insulin resistance or insufficient insulin production. Over the last thirty years, there has been a substantial surge in the occurrence of type 2 diabetes across nations of varying economic statuses. Type 1 diabetes, previously known as juvenile diabetes or insulin-dependent diabetes, is a chronic condition characterized by little to no natural insulin production by the pancreas. About 422 million individuals globally are affected by diabetes, with a majority residing in low- and middle-income nations, and an estimated 1.5 million deaths are directly linked to diabetes annually [1]. By the year 2045, International Diabetes Federation (IDF) forecasts indicate that one out of every eight adults, totaling approximately 783 million individuals, will be afflicted by diabetes, marking a surge of 46%. The vast majority, exceeding 90%, of individuals grappling with diabetes are diagnosed with type 2 diabetes, a condition propelled by a combination of socioeconomic, demographic, environmental, and genetic determinants. The primary factors contributing to the escalation of type 2 diabetes encompass urbanization, an aging populace, reduced levels of physical activity, and a growing prevalence of overweight and obesity [2]. According to data provided by the World Health Organization (WHO), the global prevalence of diabetes currently stands at approximately 450 million cases, with projections indicating a potential increase to 700 million cases by the year 2045 [3]. The population with diabetes is projected to reach 39.7 million by the year 2030 and 60.6 million by 2060 in the United States alone [4]. Furthermore, alongside the substantial count of diagnosed individuals, there exists a notable portion of the populace who remain undiagnosed due to socioeconomic and various other factors. Therefore, the emphasis on diabetes prevention has attracted increased attention internationally, especially in developed regions. Consequently, the identification and management of diabetes have evolved into a topic of considerable practical significance and economic advantages. Medical directives advocate for monitoring four times daily, with a rise to ten times per day during periods of illness or inadequate glycemic regulation [5]. Regrettably, the matter of routinely monitoring blood glucose levels is often perceived as uncomfortable by the majority of individuals with diabetes. Traditional tools employed for glucose monitoring operate based on the principles of the electrochemical method [6]. Thus the creation of an effective non-invasive glucose measurement device would be transformative for millions of patients worldwide, enabling them to monitor their glucose levels with confidence and receive prompt treatment when needed [7]. More broadly, blood glucose monitoring systems overall can be classified into two categories: invasive and non-invasive. Figure 1 shows the general classification for blood glucose monitoring, and the detailed methods are discussed in the following sections.

### 1.1. Invasive Blood Glucose Monitoring

Currently, the conventional method for blood glucose detection involves taking a blood sample or urine and analyzing it in vitro for glucose measurement. These methods are widely used in the laboratory. In hospitals, blood is drawn from patients on an empty stomach in the morning, and an automatic biochemical analyzer is used to measure the blood glucose concentration accurately. Besides blood, glucose can also be determined using urine tests [8]. Urine testing includes both non-invasive and invasive collection methods as mentioned in the general classification above. Invasive urine collection is necessary for patients who are unable to cooperate, suffer from urinary incontinence, or have external urethral ulcerations that elevate the risk of contamination [9]. Although these methods are precise and can serve as an important basis for diabetes diagnosis, they are not suitable for continuous monitoring of diabetics due to their time-consuming process, large blood sample requirement, and invasive nature. Self-monitoring of blood glucose (SMBG) is an alternative method for monitoring blood glucose concentration at a specific point in time, typically using a home electronic glucometer. These devices usually use glucose oxidase biosensors, collect fingerstick blood with a disposable strip of paper, and determine blood glucose concentration through a chemical reaction current.

The advantages of commercial glucose meters are their portability, affordability, simplicity, relatively accurate data, and ability to monitor glucose multiple times a day. However, these devices have some disadvantages, such as the need for frequent blood collection, which can lead to pain, stress, and increased risk of infection. Some common commercial glucose meter brands include Roche, Sano, Omron, Johnson and Johnson, Bayer, Abbott, Echeng, Ecco, and others [10].

### 1.2. Non-Invasive Blood Glucose Monitoring

Non-invasive blood glucose monitoring, as its name implies, involves identifying glucose levels in the bloodstream of individuals without causing harm to bodily tissues. Various techniques have been proposed and examined in recent decades. Significant effort has been invested in creating a non-invasive method for measuring glucose. Such a method would enhance the quality of life for diabetic patients and increase their adherence to regular glucose monitoring. To be considered for use in a glucose monitoring device, a method must meet the following criteria:

- Sensitivity: This denotes the minimum concentration that a sensor can detect. A blood glucose sensor should be capable of identifying glucose levels as low as 30 mg/dL [11].
- Stability: This pertains to the performance of a measurement device over an extended period. The device should exhibit high precision, ensuring that measurements remain consistent for the same concentration. Additionally, it should offer a high level of accuracy, meaning that measurements should not fluctuate over time.
- Selectivity: The measurement method must be able to distinguish the glucose signal from signals generated by other substances. Since glucose in the human body is present in aqueous solutions that also contain ions or proteins, which could produce interfering signals, the sensor must effectively isolate the glucose signal.
- Portability: The measurement device should be compact and convenient to carry.

Key parameters for non-invasive glucose monitoring devices include selectivity, stability, portability, and sensitivity. Stability ensures consistent performance, while selectivity allows accurate glucose detection in the presence of other substances. Portability makes the device practical for everyday use, and sensitivity ensures that it can detect small changes in glucose levels. These factors are vital for developing effective and user-friendly monitoring systems.

Due to the difficulty of directly accessing blood for non-invasive glucose measurement, alternative biofluids such as urine [12], saliva [13], tears [14], and sweat [15] have been explored. However, glucose levels in these fluids often show a weak correlation with blood glucose levels [16] and experience a significant lag time [17], making them less viable. For instance, saliva glucose measurements can be influenced by pH changes after consuming acidic foods. A promising alternative is to measure glucose concentration from the interstitial fluid (ISF) in the epidermal layer of the skin because ISF constitutes the extracellular fluid that surrounds tissue cells and is composed of numerous significant biomarkers (biomolecules present in blood, other bodily fluids, or tissues that indicate a normal or abnormal process or a condition or disease [18]), exhibiting comparable medical diagnostic capabilities to that of blood. Minor molecular biomarkers undergo exchange between blood and ISF via the process of diffusion. Consequently, the relationship between ISF and blood may be employed to indirectly acquire health-related information about patients [10]. The market for non-invasive blood glucose monitoring systems is expected to grow significantly, from USD 21.76 million in 2022 to an estimated USD 202.73 million by 2032, with a compound annual growth rate (CAGR) of 25.0%. This growth is largely due to the increasing prevalence of diabetes and the frequent complications associated with the disease [19].

Non-invasive methods can be broadly classified as either optical or non-optical methods. There are several non-optical approaches, as shown in Figure 1, such as impedance spectroscopy [20], electromagnetic [21], reverse iontophoresis [22], metabolic heat confirmation [23], and ultrasonic approaches [24]. This methodology possesses the capability to modify the characteristics of the dermal layer and provoke phenomena such as blister formation, irritation, or erythema [20]. Furthermore, this technique necessitates meticulous instrumentation and rigorous calibration, which can be labor-intensive and challenging to execute. Optical spectroscopy is less likely to cause skin irritation compared to non-optical methods and demonstrates a high level of specificity for detecting glucose, even within complex matrices such as blood [25]. Consequently, this review emphasizes and discusses optical methods in detail. Some of them are fluorescence spectroscopy [26,27], optical coherence tomography [28], thermal spectroscopy [29,30], diffuse reflectance spectroscopy [31,32], polarimetry [33,34], absorption spectroscopy [35,36], and photoacoustic spectroscopy [37,38]. In recent years, photoacoustic spectroscopy (PAS) has demonstrated effectiveness in non-invasive glucose detection due to its increased sensitivity compared to optical absorption spectroscopy [39,40,41]. The photoacoustic effect has shown promise in a range of applications for both gaseous and solid samples [42].

The PAS process generally employs infrared laser radiation to excite the vibrational states of molecules. Because the radiation is modulated, the heat released by the molecules is also modulated, causing periodic pressure fluctuations in the surrounding environment, which are detected as acoustic waves by a microphone or acoustic transducer. The generated acoustic signal is typically weak and requires amplification before further processing. This can be accomplished by using an acoustic resonator, where corresponding acoustic modes are excited by the laser illumination. This approach significantly enhances the photoacoustic signal and improves the sensor’s overall detection sensitivity. To maximize signal amplification and neglect some of the environmental factors that deteriorate the actual signal, the resonator’s shape must be optimized.

This study provides an overview of the photoacoustic resonators (PARs) that are used in PAS for non-invasive blood glucose detection. Here, we describe the basic principle of PAS with an emphasis on PARs for acoustic signal amplification, where the amplification mechanism and performance in the context of non-invasive glucose monitoring are thoroughly highlighted based on studies published in the literature from 2012 to 2023. The main motivation here is to identify and analyze the key parameters essential for the effective design of PARs used in non-invasive glucose detection. This work is presented in the following manner: Section 2 covers the fundamental principles and physics of conventional photoacoustic spectroscopy (PAS). It also explains how photoacoustic resonance (PAR) amplifies signals, along with the design and modeling techniques involved, as well as the factors that affect quality. In Section 3, a brief history of the use of photoacoustic cells for non-invasive glucose detection from 2012 to 2023 is provided, along with a review of the key literature published during that period. Lastly, Section 4 discusses potential future developments in the use of PAR for non-invasive glucose detection. After analyzing various designs and their performance parameters, we aim to provide guidance on how factors such as resonance frequency, quality factor, and geometry impact the sensitivity of PARs.

## 2. Principle of Photoacoustic Spectroscopy for Non-Invasive Glucose Detection

### 2.1. Basic Interaction of IR Light with Human Skin/Tissue

**Figure 2:**
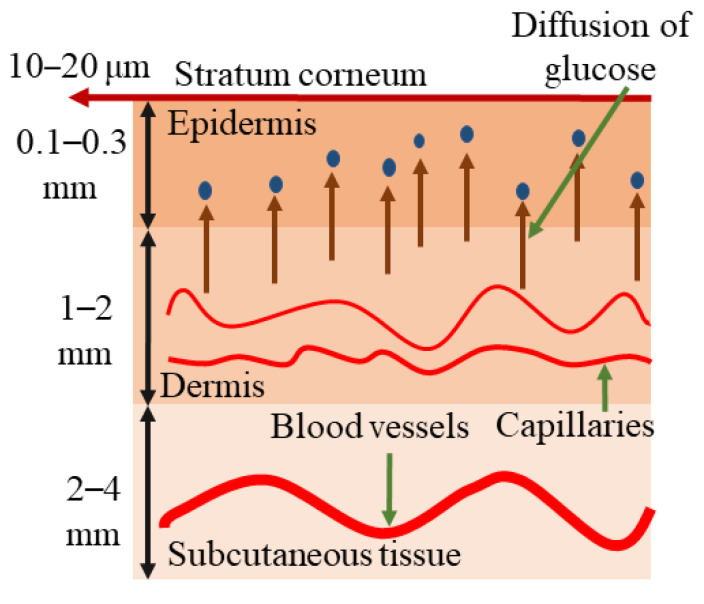
Schematic representation of the components of human skin [43].

Figure 2 exhibits a schematic of typical human skin, which starts with the layer known as the stratum corneum (the outmost layer of epidermis). The skin consists mainly of three layers: the epidermis, the dermis, and the subcutaneous tissue [43] As the PAS combines optical spectroscopy and ultrasound tomography, an optical source needs to be selected that is sensitive to glucose molecules. Mostly, infrared (IR) spectroscopy examines the interaction between tissue and infrared radiation, with wavelengths ranging from 700 nm to 25,000 nm in the electromagnetic spectrum. This technique is rooted in vibrational spectroscopy and provides a quick method for both qualitative and quantitative analyses through direct measurement. For improved analytical capabilities, the IR spectrum is divided into three regions: near-infrared (NIR) from 700 to 2500 nm, mid-infrared (MIR) from 2500 to 25,000 nm, and far-infrared (FIR) from 25,000 to 1,000,000 nm [44].

**Figure 3:**
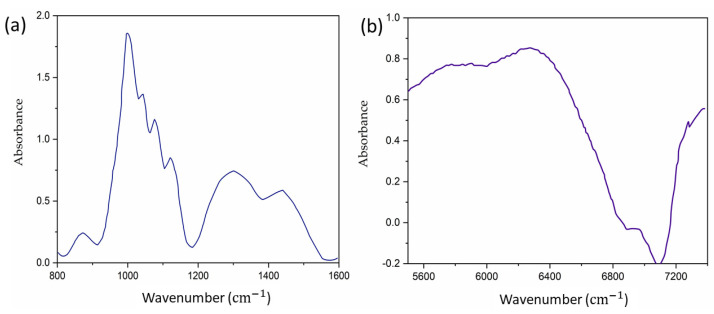
(**a**) Absorption spectrum for aqueous glucose in the MIR region extending from 930 cm^−1^ to 1200 cm^−1^ [17]. (**b**) Absorption spectrum of glucose in the NIR region from 5550 cm^−1^ to 7400 cm^−1^ [50].

NIR and MIR light sources are generally explored as excitation sources due to the strong glucose signature in these regions. Figure 3a shows the absorption spectra of aqueous glucose in the MIR region, with water absorption subtracted from the spectra. Glucose absorption peaks are identified at approximately 920 cm^−1^, 1090 cm^−1^, 1120 cm^−1^, and 1140 cm^−1^. On the other hand, Figure 3b represents the absorption spectrum of glucose for the NIR region ranging from 5550 cm^−1^ to 7400 cm^−1^. NIR measurements examine harmonic overtones of glucose vibrations [45], resulting in relatively weak glucose absorption compared to the background water absorption. The accuracy of these measurements is hindered by significant interference from tissue and other blood components with similar absorption spectra [46,47]. Additionally, NIR measurements are influenced by strong tissue scattering, unlike mid-infrared light measurements. MIR light is strongly absorbed by glucose molecules at wavelengths between 8 and 10 µm due to the fundamental vibrational resonances [25,48]. The acoustic signal that is generated can go through human tissue with minimal scattering and be detected by a microphone/transducer. Thus, the glucose information in the interstitial fluid (ISF) can be correlated with the acoustic signal in IR-based PAS [48]. The ISF is a layer of biological fluid found between cells and consists of water, solvents, and blood vessels. It contains various substances such as sugars, fats, amino acids, hormones, coenzymes, white blood cells, and cellular waste products. Glucose is known to diffuse from the blood to the ISF layers, with a delay of 5 to 15 min [49,50].

### 2.2. Principle of Generating and Detecting Photoacoustic Signals

**Figure 4:**
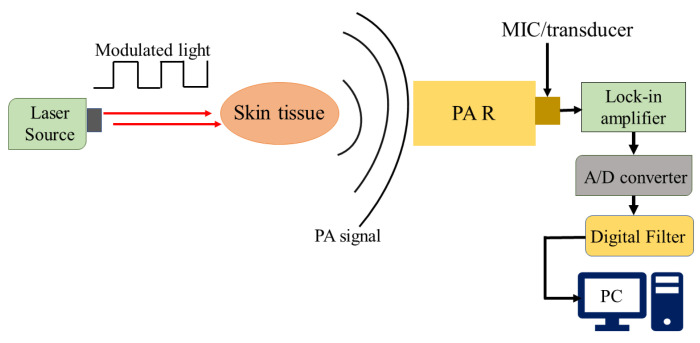
The basic experimental setup for the generation and detection of acoustic signal used in PAS (MIC: microphone; PAR: photoacoustic resonator; A/D converter: analog-to-digital converter).

Figure 4 shows the simplified schematic representation of the basic experimental setup of PAS for the detection of non-invasive blood glucose. The concept of PAS entails the initiation of acoustical waves via a pulsed electromagnetic source (e.g., IR lasers). The biological entity absorbs these electromagnetic waves, inducing thermal expansion or pressure, thereby giving rise to the generation of acoustical waves [51]. The signal generated by the photoacoustic effect is typically weak and requires amplification before further processing. This can be accomplished by adapting a photoacoustic resonator (PAR) in the setup, such that the electromagnetic radiation is modulated at the PAR’s acoustic eigenfrequency, thereby exciting the corresponding acoustic mode. This approach significantly enhances the photoacoustic signal and improves the sensor’s detection sensitivity. Optimizing the resonator’s design parameters and using suitable filters can further maximize the amplification and detection sensitivity of the photoacoustic signal. Subsequently, sensitive ultrasonic microphones or piezoelectric transducers are capable of capturing the amplified waves, which are fed to a lock-in amplifier allowing noise reduction and further processing. Furthermore, the attributes of the modulated light source, such as modulation amplitude and duty cycle, exert a notable influence on the production of acoustical waves. The characteristics of human skin play a vital role in the process of wave generation. The pressure resulting from the photoacoustic phenomenon can be mathematically described by the following wave equation incorporating spatial heat distribution *H*(*r*, *t*), following the principles of thermodynamics [51]

(1) $$\nabla^{2}p\left(r,t\right)-\frac{1}{v^{2}}\frac{\delta^{2}}{\delta t}p\left(r,t\right)=-\frac{\beta }{C_{p}}\;\frac{\delta }{\delta t}H\left(r,t\right)\;$$

where $p\left(r,t\right)$ represents spatial pressure distribution at time *t*, *v* is the acoustic velocity, *Cp* is the heat capacity, and *β* is the thermal expansion coefficient.

The equation below can be used to represent the peak pressure (*P*) for a medium that absorbs light weakly:

(2) $$P=k\;\frac{\beta v^{n}}{C_{p}}\;E_{0}\alpha \;$$

The system constant, denoted as *k*, is present in this context, alongside $E_{0}$ representing the incident laser pulse energy, and *n*, a constant falling within the range of 1 to 2, based on the specific experimental setup. At times, the combination of experimental variables may result in a heightened photoacoustic signal in contrast to conventional spectroscopic approaches. It is crucial to highlight that the response generated by glucose lacks the requisite level of signal amplification for detection purposes, necessitating the utilization of a PAR to amplify the signal.

### 2.3. Amplification Mechanism of Photoacoustic Resonator (PAR)

**Figure 5:**
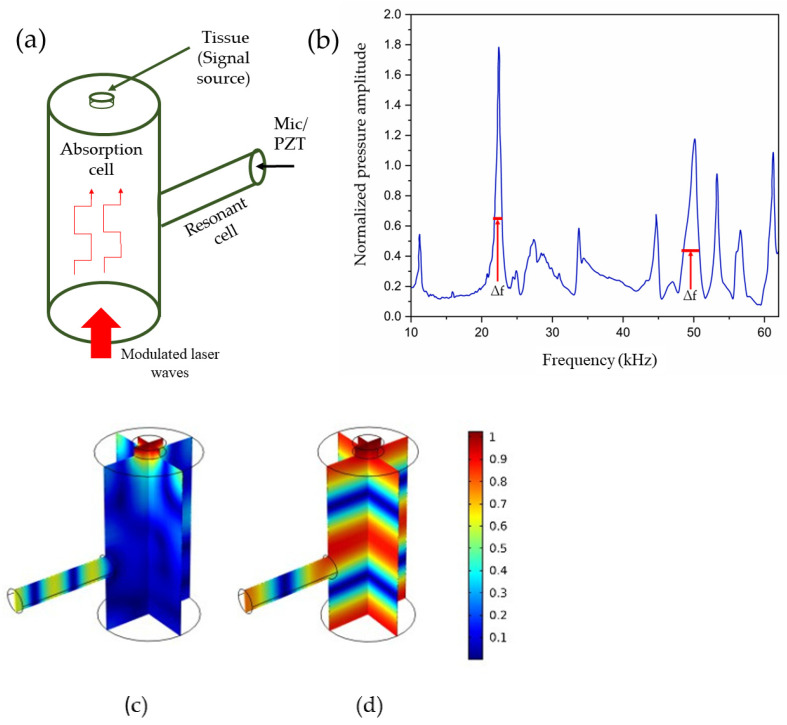
(**a**) A schematic representation of a typical T-type PAR. (**b**) The frequency response of a PAR. (**c**,**d**) A profile of acoustic modes at the peak frequencies of 49.5 kHz and 22.2 kHz, respectively, corresponding to (**b**) [52].

The signal generated by the photoacoustic effect tends to be relatively weak and necessitates amplification. This amplification can be accomplished by modulating the electromagnetic waves at an acoustic resonant frequency of a photoacoustic resonator, thereby stimulating the corresponding acoustic mode. “Acoustic modes” are the specific patterns of pressure variations or sound waves that resonate within a confined space, for example, a resonator, at certain frequencies. These modes are influenced by the resonator’s size and shape and by the properties of the medium inside it (such as air). Each mode corresponds to a particular frequency, known as the eigenfrequency or resonant frequency, where the resonator naturally enhances sound waves. Exciting these acoustic modes can significantly boost the signal strength of effects such as the photoacoustic effect, thereby improving detection sensitivity in various applications [52]. Figure 5a shows a schematic representation of a typical T-type PAR, where two cells are combined: absorption and resonant.

The quality factor is considered to be the performance-indicating parameter of an acoustic resonator and can be defined as the ratio of the resonance frequency to the bandwidth of the resonance peak. A higher quality factor (*Q*) implies a sharper resonance peak and can be represented as follows:

(3) $$Q=\frac{f}{∆f}\;\;\;\;$$

where *f* denotes the resonance frequency, while ∆f is the difference in frequencies at which the value of the pressure amplitude has decreased to half of the resonance value. Enhancing the shape of the resonator can further optimize the amplification of the photoacoustic signal, as it enhances the quality factor by decreasing ∆f.

### 2.4. Design and Modeling of Photoacoustic Resonator

In general, the modeling of a PAR can be achieved in various ways: experimental modeling and numerical modeling [52], quantitative modeling [53] optoacoustic inversion modeling [54], structural and functional modeling [55], the finite element method [56], etc. When conducting a purely experimental study, it becomes necessary to experiment with various resonator configurations, as determining the most suitable geometry for maximal signal enhancement is not straightforward. This undertaking may prove to be exceedingly laborious and costly. Consequently, computational simulation techniques are the preferred approach [57,58,59]. El-Busaidy et al. [52] described two simulation models known as the VT (viscothermal) and AME (amplitude mode expansion) models. The VT model is widely regarded as the most precise numerical method for simulating photoacoustic signals in a PAR due to its ability to accurately represent loss effects at the resonator surfaces, which are the primary loss mechanisms in PARs [58]. However, the VT model is computationally intensive, requiring significant memory and simulation time; for that reason, they investigated the AME model, which is faster and computationally less demanding [59], for a closed T-cell resonator. They expanded the study of the AME model by simulating the photoacoustic signal of a macroscopic T-cell over a broad frequency range, from 8 kHz to 62 kHz. Their focus was on the photoacoustic signal generated by a solid sample, rather than a gaseous one. The solid sample was positioned at one of the resonator’s openings, with the resonator being filled with air instead of a gaseous absorbing sample. Measurements of the photoacoustic signal from the simulated resonator were conducted and then compared to the simulated results [52].

**Figure 6:**
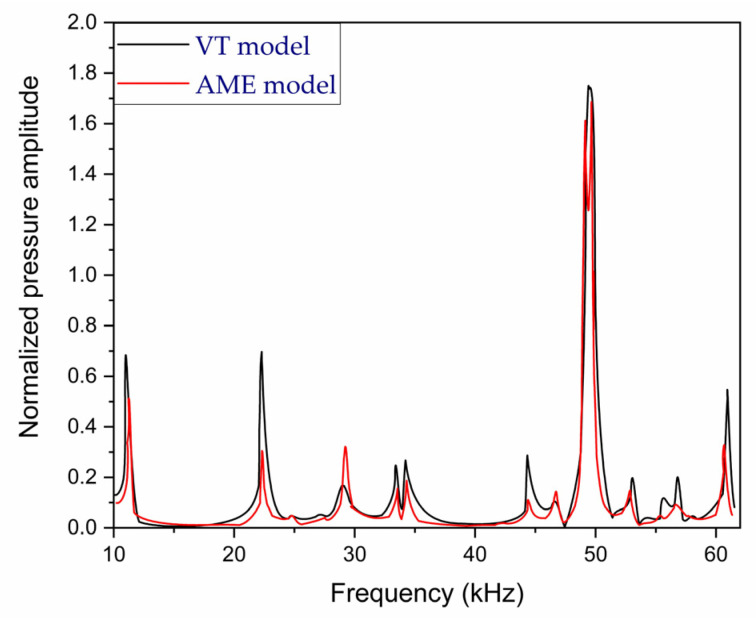
The frequency responses of the amplitude mode expansion (AME) model and the viscothermal (VT) model [52].

Figure 6 shows two simulated frequency–response curves between 10 kHz and 62 kHz using VT and AME simulation models. The AME model’s measurement results were compared to those of the VT model, which is regarded as the most accurate simulation method. The resonance frequencies from the measurements showed good accordance with the VT simulations, with a relative difference in peak resonance frequency of not more than 1.1% compared to the VT model values. It should be noted that the temperature of the air inside the resonator was not monitored during the measurements. Temperature variations could contribute to deviations, as the speed of sound and, consequently, the resonance frequencies are dependent on the air temperature [52].

### 2.5. Requirements of PAR for Non-Invasive Glucose Detection

The key aspects to consider when developing a PAR system for non-invasive glucose detection include [56] the following:

- Humidity issue:

One of the ends is left unsealed to prevent an increase in humidity within the resonator caused by skin transpiration [60]; this is the reason why photoacoustic measurements of blood glucose levels are performed using open T-shaped resonators [60,61]. Furthermore, the opening enhances the stability of the measurement by reducing the temperature fluctuations [61].

- Volume and surface loss:

If the thermal conductivity of the fluid (i.e., air inside the cell) is negligible, there is no heat transfer between adjacent pressure maxima and minima. However, if this heat transfer cannot be ignored, the fluctuations in energy density will diminish, leading to the dissipation of the sound wave.

- Location and position of light source and cylinders:

The location of laser beams in the T-cell and the position of the resonance cylinder and the microphones also define the performance of PAR. The additional loss is due to leakage of the PA signal from the resonator end sealed with the microphone [62].

- Signal-to-noise ratio:

Obtaining a proper signal-to-noise ratio (SNR) is essential for improving the accuracy of non-invasive glucose detection. Pleitz et al. (2013) [41] demonstrated the use of a quantum cascade laser with an optimized photoacoustic setup that significantly reduces the noise from external sources, allowing more accurate glucose readings from the skin and minimizing background noise from other skin components, ultimately enhancing the system’s reliability and sensitivity.

## 3. History of PA Cells Used in PAS for Non-Invasive Blood Glucose Detection

Camou et al. [63] designed a detection photoacoustic (PA) cell in 2012 that is made of brass and provides a sufficient acoustic impedance mismatch with water so that acoustic energy remains confined within the inner volume of the cell. In their study, they used a piezoelectric transducer that was set perpendicular to the optical fiber so that the light beam could not directly strike the sensitive surface of the transducer. This cylindrical, fiber-coupled PA cell’s working frequency range was 300–500 kHz, and they used aqueous glucose solutions whose concentration ranged from 0 to 15 g/dL. In 2013, the same research group [64] proposed a fabrication method that consisted of patterning three PMMA plates with a laser cutter to ensure fast prototyping and flexibility in the design. They used the same frequency range of 300–500 kHz, and the glucose solution concentration levels ranged from 0 to 10 g/dL.

Pleitez et al. [41] designed a T-shaped PA cell for in vivo measurement of blood glucose. This combination facilitates a quantitative measurement for concentrations of skin glucose in the range from <50 mg/dL to >300 mg/dL. This range is relevant for glucose monitoring in diabetes patients. In the T-shaped resonator, there existed two perpendicularly connected cylindrical cavities, which are the absorption and resonance cavities. An anti-reflection-coated ZnSe window was included for antireflection purposes. This T-shaped PA cell had a working frequency range of 50–54 kHz with a quality (Q) factor of 102. Later on, the same researchers [60] showed the application of a novel open, windowless cell in 2013 for photoacoustic IR spectroscopy of human skin. The windowless cavity was tuned for optimum performance in the ultrasound range between 50–60 kHz. In that study, they used open cells to prevent an increase in humidity inside the acoustic resonator due to skin transpiration. The frequency response of the open cell was found to be dominated by two resonance peaks at 51.7 kHz and 53.8 kHz having quality factors of 45 and 32, respectively. Finally, they observed that the Q factor for the same windowless PA cell was about 50% lower than the Q factor for the same PA cell geometry in the closed configuration.

Kottmann et al. [40] presented the implementation of the first fiber-coupled MIR photoacoustic sensor for the investigation of condensed samples in the MIR fingerprint region. The PA chamber was conical in shape to perfectly match the beam escaping the fiber and to minimize the cell volume. This setup yielded a detection limit of 57 mg/dL with SNR = 1. This lies within the physiological range (i.e., 30–500 mg/dL), but it needs to be improved if non-invasive in vivo glucose measurements are to become feasible in the future. In 2016, the same research group [43] presented a new study based on MIR spectroscopy and PA detection. In that study, they employed two setups. One was a fiber-coupled PA cell with a tunable QCL (quantum cascade laser), and the other consisted of two QCLs at different wavelengths combined with PA detection. Finally, they conducted a performance test with an OGTT (oral glucose tolerance test). The PA cell was conical, and the range of glucose concentrations used was 0–440 mg/dL.

In 2015 [65], Wadamori et al. showed a configuration of PA cells that were used in conjunction with the sample reservoir to monitor time-dependent changes in the concentration of glucose dissolved in distilled water. Each PA signal was obtained by averaging (1000 times) the PA signal at a modulation frequency of 5.05 kHz, which was a resonance frequency for the cell. The resonator was T-shaped, and the glucose concentration was measured in a range of 30–500 mg/dL.

In 2016, Sim et al. [66] depicted a way of increasing the sensitivity of the PAS system by crossmatching a resonance peak of their PA cell with a microphone. Their cell was T-shaped, consisting of two intersecting cylinders, allowing independent optimization of key parameters to determine the signal strength. The resonance frequency of their designed cell was 51 kHz, and the concentration of used glucose was 1000 mg/dL. In 2018, the same research group [61] exhibited a T-shaped PA cell having a resonant frequency of 47.5 kHz. They presented a method to meet the challenges of MIR photoacoustic spectroscopy by obtaining microscopic spatial information about the skin during the spectroscopy measurement; a skin region where the IR spectra were insensitive to skin condition could be locally selected, enabling a reliable prognosis of the glucose level from the PAS signal.

Zhao et al. [67] in the year 2017, demonstrated the theory of liquid photoacoustic resonance by giving a rigorous mathematical expression. A signal processing method was also exhibited at the same time under liquid PA resonance conditions. The feasibility and validity were also verified through experiments with different concentrations of glucose solution. This method overcame the issue of lack of sensitivity and inaccurate detection in the non-resonant case and produced accurate results. The cylindrical PA had has an excitation wavelength of 1064 nm, and the transducer that was used had a peak resonant frequency of 310 kHz; the range of glucose concentration used here was 0–440 mg/dL.

El-Busaidy et al. [68] presented two new approaches for simulating the PA signal in an open PA resonator using finite elements. The approaches were the amplitude mode expansion model and the viscothermal model. They verified the simulation results by comparing them to PA measurements. These methods provide an accurate basis for designing and optimizing open resonators with high sensitivity.

Yang et al. [69] in 2022 designed a bowl-shaped PA cell structure, which used a focused ultrasonic transducer to receive signals. In their setup, the ultrasonic transducer was immersed in the liquid, and the PA signal was generated and propagated directly in the liquid. Compared with air, the attenuation of ultrasonic signal in liquid is very small; therefore, this device could ensure good signal accuracy.

Aloraynan et al. [70] in 2023 developed a dual single-wavelength QCL-based system using photoacoustic spectroscopy for non-invasive glucose measurement. They developed biomedical skin phantoms with properties quite similar to those of human skin. The detection sensitivity of their developed system was 12.5 mg/dL. Finally, they developed a machine learning model for further analysis. The PA cell that they used was T-shaped, and the range of glucose concentrations used was 100–275 mg/dL.

Table 1 summarizes the latest progress in non-invasive glucose sensing using infrared PAS. It presents key details such as the year of publication, the type of photoacoustic cell (PAC) used, the modulation frequency range, the quality factor, and the glucose level tested. The table highlights the different designs and approaches researchers have employed over time, such as using cylindrical or T-shaped cells, to improve sensitivity in glucose detection. The overview gives a clear pictorial representation of how PAS technology has advanced with the effective design of PACs and how these developments are helping to make non-invasive glucose monitoring more effective.

## 4. Prospects of PARs for Non-Invasive Glucose Detection

The strength of the photoacoustic signal is strongly influenced by resonance amplification, and numerous studies have focused on optimizing PA sensors. Building on these insights, further improvements are necessary to achieve optimal performance. The following factors could be considered for designing highly sensitive PARs for non-invasive glucose detection.

- Finding proper geometry:

Detection sensitivity is significantly influenced by the geometric shape of the resonator. For example, in one paper [71], the authors demonstrated that the intensity of the generated PA signal is inversely proportional to the volume of a non-resonant cell. High detection sensitivity can be achieved by using a small-sized Helmholtz resonant cell. In contrast, conventional straight-type resonant cells, which have a large dead volume, result in lower detection sensitivity [72]. Thus, to achieve an effective design, the appropriate geometry should be selected, taking into account the factors mentioned above.

- Identifying proper resonant frequency of cell:

The resonant frequency of the cell should be determined through numerical acoustic analysis, employing methods such as the finite element method. Since the resonant frequency of the resonator is the most sensitive and provides the greatest amplification, it is crucial to account for acoustic losses, including viscosity and heat exchange within the cell, to ensure proper functioning [72].

- Observation of detection sensitivity and signal-to-noise ratio (SNR):

Since tissues contain various fluids, primarily water, which creates background noise, a designer needs to focus on calculating the signal-to-noise ratio (SNR) that can be achieved with the resonator. For instance, the SNR of a small Helmholtz-type resonant cell was found to be approximately 69 dB, compared to 62 dB [72] for a conventional straight-type resonant cell. Both of these values are significantly higher than the required SNR of 50 dB [73,74].

## 5. Conclusions

The purpose of this review was to explore and highlight essential factors that influence the effective design of PARs for non-invasive glucose monitoring. In this review, we concentrated on the recent advancements in the development of photoacoustic resonators, which are crucial for non-invasive glucose measurement using photoacoustic spectroscopy (PAS) in the infrared (IR) spectrum, particularly in the mid-infrared (MIR) and near-infrared (NIR) regions. PAS shows significant promise for future non-invasive techniques. The photoacoustic resonator is essential for amplifying specific modes, as the signals generated by the photoacoustic effect are inherently weak and require appropriate amplification. This work investigated and reviewed optimal resonator geometries and their performance. We also discussed the primary limitations and identified the main sources of losses in resonators. Among various simulation models, we focused on the viscothermal model and the amplitude mode expansion model, as discussed in the existing literature. Finally, we highlighted the potential of IR-based PAS for non-invasive blood glucose detection, emphasizing that using a well-designed resonator could lead to more desirable outcomes. Some key technical challenges include managing humidity within the resonator to maintain stable measurements, minimizing volume and surface loss, and optimizing the signal-to-noise ratio (SNR) to ensure accurate non-invasive glucose detection amidst external noise. These shortcomings can be mitigated by optimizing the geometry of the resonator, such as using smaller open Helmholtz resonant cells which can significantly increase sensitivity, additionally, improving the signal-to-noise ratio (SNR) by reducing background noise, which will help to achieve more accurate glucose measurements. Finally, this review can serve as a key reference for future research on the development of optimal photoacoustic resonators, contributing to the accurate and reliable detection of glucose.

## Figures and Tables

**Table 1:** A detailed summary of the recent progress in non-invasive glucose sensing using MIR-based PAS. PAC: photoacoustic cell; QCL: quantum cascade laser; PA: photoacoustic; EC: external cavity.

Year of Publication	Excitation Wavelength (nm)	Type of PA Cell	Frequency Range (kHz)	Q-Factor	Investigated Sample	Glucose Level (mg/dL)	Schematic of PA Cell
2012 [63]	1382 and 1610	Cylindrical Fiber-coupled	300–500	-----	Aqueous glucose solution	50, 100, 150	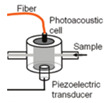
2012 [41]	8196 to 10,000	T-shaped	50–54	102	Fingertips of healthy and diabetes affected volunteers	<50, and <300	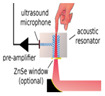
2013 [64]	1382 to 1610	Cylindrical Fiber-coupled	300–500	------	Aqueous glucose solution	0–100	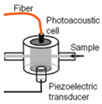
2013 [40]	9090 to 9132	Conical	------	-------	Both in aqueous glucose solution and different body sites of human	30–500	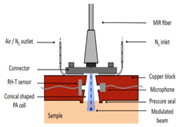
2013 [60]	8032 to 10,000	T-shaped	50–60	------	Fingertips of healthy and diabetes-affected volunteers	30–500	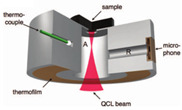
2015 [65]	1550	T-shaped	5.05 (resonance frequency of PA cell)	------	Aqueous glucose solution	30–500	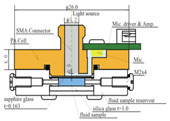
2016 [66]	8064 to 11,111	T-shaped	51 (resonance frequency of PA cell)	------	Carbon black tape as reference sample	1000	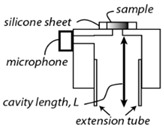
2016 [43]	8032 to 10,000	Conical	-------	-------	Both aqueous glucose solution and human fingertips	0–440	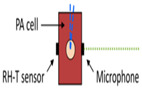
2017 [67]	1064	Cylindrical	310 (resonant peak of PZT)	------	Aqueous glucose solution	20–100	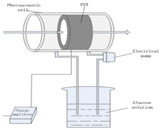
2018 [61]	8000 to 11,111	T-shaped	47.5 (resonant peak of PA cell)	------	Index fingertip	------	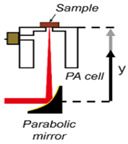
2020 [68]	-----	T-shaped	10–60	-----	------	-----	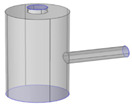
2022 [69]	1535	Bowl-shaped	0–5 (for bandpass signal processing)	------	Aqueous glucose solution	30 to 500	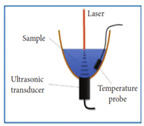
2023 [70]	9250	T-shaped	10 to 40 (with a frequency step of 0.15 kHz)	------	Biomedical skin phantom	100 to 275	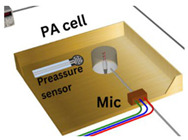

## Data Availability

Not applicable.

## References

[1] Diabetes. https://www.who.int/health-topics/diabetes?gad_source=1#tab=tab_1.

[2] Diabetes Facts and Figures|International Diabetes Federation. https://idf.org/about-diabetes/diabetes-facts-figures/.

[3] Cho N.H., Shaw J.E., Karuranga S., Huang Y., da Rocha Fernandes J.D., Ohlrogge A.W., Malanda B. (2018). IDF Diabetes Atlas: Global estimates of diabetes prevalence for 2017 and projections for 2045. Diabetes Res. Clin. Pract..

[4] Lin J., Thompson T.J., Cheng Y.J., Zhuo X., Zhang P., Gregg E., Rolka D.B. (2018). Projection of the future diabetes burden in the United States through 2060. Popul. Health Metr..

[5] (2010). Standards of Medical Care in Diabetes—2010. Diabetes Care.

[6] Clark L.C., Lyons C. (1962). ELECTRODE SYSTEMS FOR CONTINUOUS MONITORING IN CARDIOVASCULAR SURGERY. Ann. N. Y. Acad. Sci..

[7] Villena Gonzales W., Mobashsher A., Abbosh A. (2019). The Progress of Glucose Monitoring—A Review of Invasive to Minimally and Non-Invasive Techniques, Devices and Sensors. Sensors.

[8] Phenobarbital—Health Encyclopedia—University of Rochester Medical Center 2017. https://www.urmc.rochester.edu/encyclopedia/content.aspx?ContentTypeID=167&ContentID=glucose_urine.

[9] Ross D.G. (2023). Urinalysis. Imaging and Technology in Urology.

[10] Tang L., Chang S.J., Chen C.-J., Liu J.-T. (2020). Non-Invasive Blood Glucose Monitoring Technology: A Review. Sensors.

[11] von Lilienfeld-Toal H., Weidenmüller M., Xhelaj A., Mäntele W. (2005). A novel approach to non-invasive glucose measurement by mid-infrared spectroscopy: The combination of quantum cascade lasers (QCL) and photoacoustic detection. Vib. Spectrosc..

[12] Park H.D., Lee K.J., Yoon H.R., Nam H.H. (2005). Design of a portable urine glucose monitoring system for health care. Comput. Biol. Med..

[13] Panchbhai A.S. (2012). Correlation of Salivary Glucose Level with Blood Glucose Level in Diabetes Mellitus. J. Oral Maxillofac. Res..

[14] Iguchi S., Kudo H., Saito T., Ogawa M., Saito H., Otsuka K., Funakubo A., Mitsubayashi K. (2007). A flexible and wearable biosensor for tear glucose measurement. Biomed. Microdevices.

[15] Moyer J., Wilson D., Finkelshtein I., Wong B., Potts R. (2012). Correlation Between Sweat Glucose and Blood Glucose in Subjects with Diabetes. Diabetes Technol. Ther..

[16] Delbeck S., Vahlsing T., Leonhardt S., Steiner G., Heise H.M. (2019). Non-invasive monitoring of blood glucose using optical methods for skin spectroscopy—Opportunities and recent advances. Anal. Bioanal. Chem..

[17] Pleitez M., von Lilienfeld-Toal H., Mäntele W. (2012). Infrared spectroscopic analysis of human interstitial fluid in vitro and in vivo using FT-IR spectroscopy and pulsed quantum cascade lasers (QCL): Establishing a new approach to non invasive glucose measurement. Spectrochim. Acta Part A Mol. Biomol. Spectrosc..

[18] Definition of biomarker—NCI Dictionary of Cancer Terms—NCI. https://www.cancer.gov/publications/dictionaries/cancer-terms/def/biomarker.

[19] Non-Invasive Blood Glucose Monitoring System Market Size Envisioned at USD 202.73 Million by 2032. https://www.towardshealthcare.com/insights/non-invasive-blood-glucose-monitoring-system-market.

[20] Huang J., Zhang Y., Wu J. (2020). Review of non-invasive continuous glucose monitoring based on impedance spectroscopy. Sens. Actuators A Phys..

[21] Buehler L.A., Balasubramanian V., Baskerville S., Bailey R., McCarthy K., Rippen M., Bena J.F., Lansang M.C. (2022). Noninvasive Glucose Monitor Using Dielectric Spectroscopy. Endocr. Pract..

[22] Sieg A., Guy R.H., Delgado-Charro M.B. (2004). Noninvasive Glucose Monitoring by Reverse Iontophoresis in Vivo: Application of the Internal Standard Concept. Clin. Chem..

[23] Tang F., Wang X., Wang D., Li J. (2008). Non-Invasive Glucose Measurement by Use of Metabolic Heat Conformation Method. Sensors.

[24] Kost J. (2002). Ultrasound-Assisted Insulin Delivery and Noninvasive Glucose Sensing. Diabetes Technol. Ther..

[25] Kaysir M.R., Song J., Rassel S., Aloraynan A., Ban D. (2023). Progress and Perspectives of Mid-Infrared Photoacoustic Spectroscopy for Non-Invasive Glucose Detection. Biosensors.

[26] Ballerstadt R., Evans C., Gowda A., McNichols R. (2006). In Vivo Performance Evaluation of a Transdermal Near- Infrared Fluorescence Resonance Energy Transfer Affinity Sensor for Continuous Glucose Monitoring. Diabetes Technol. Ther..

[27] March W., Lazzaro D., Rastogi S. (2006). Fluorescent Measurement in the Non-Invasive Contact Lens Glucose Sensor. Diabetes Technol. Ther..

[28] Esenaliev R.O., Larin K.V., Larina I.V., Motamedi M. (2001). Noninvasive monitoring of glucose concentration with optical coherence tomography. Opt. Lett..

[29] Enejder A.M.K., Scecina T.G., Oh J., Hunter M., Shih W.-C., Sasic S., Horowitz G.L., Feld M.S. (2005). Raman spectroscopy for noninvasive glucose measurements. J. Biomed. Opt..

[30] Lambert J.L., Pelletier C.C., Borchert M. (2005). Glucose determination in human aqueous humor with Raman spectroscopy. J. Biomed. Opt..

[31] Maruo K., Tsurugi M., Tamura M., Ozaki Y. (2003). In Vivo Noninvasive Measurement of Blood Glucose by Near-Infrared Diffuse-Reflectance Spectroscopy. Appl. Spectrosc..

[32] Marbach R., Koschinsky T., Gries F.A., Heise H.M. (1993). Noninvasive Blood Glucose Assay by Near-Infrared Diffuse Reflectance Spectroscopy of the Human Inner Lip. Appl. Spectrosc..

[33] Malik B.H., Coté G.L. (2010). Real-time, closed-loop dual-wavelength optical polarimetry for glucose monitoring. J. Biomed. Opt..

[34] Purvinis G., Cameron B.D., Altrogge D.M. (2011). Noninvasive Polarimetric-Based Glucose Monitoring: An in Vivo Study. J. Diabetes Sci. Technol..

[35] Vrančić C., Fomichova A., Gretz N., Herrmann C., Neudecker S., Pucci A., Petrich W. (2011). Continuous glucose monitoring by means of mid-infrared transmission laser spectroscopy in vitro. Analyst.

[36] Spanner G., Nießner R. (1996). New concept for the non-invasive determination of physiological glucose concentrations using modulated laser diodes. Anal. Bioanal. Chem..

[37] Kottmann J., Rey J.M., Sigrist M.W. (2011). New photoacoustic cell design for studying aqueous solutions and gels. Rev. Sci. Instrum..

[38] Spanner G., Niessner R. (1996). Noninvasive determination of blood constituents using an array of modulated laser diodes and a photoacoustic sensor head. Anal. Bioanal. Chem..

[39] Pai P.P., Sanki P.K., Banerjee S. (2015). A photoacoustics based continuous non-invasive blood glucose monitoring system. Proceedings of the 2015 IEEE International Symposium on Medical Measurements and Applications (MeMeA) Proceedings.

[40] Kottmann J., Grob U., Rey J., Sigrist M. (2013). Mid-Infrared Fiber-Coupled Photoacoustic Sensor for Biomedical Applications. Sensors.

[41] Pleitez M.A., Lieblein T., Bauer A., Hertzberg O., von Lilienfeld-Toal H., Mäntele W. (2013). In Vivo Noninvasive Monitoring of Glucose Concentration in Human Epidermis by Mid-Infrared Pulsed Photoacoustic Spectroscopy. Anal. Chem..

[42] Beard P. (2011). Biomedical photoacoustic imaging. Interface Focus.

[43] Kottmann J., Rey J.M., Sigrist M.W. (2016). Mid-Infrared Photoacoustic Detection of Glucose in Human Skin: Towards Non-Invasive Diagnostics. Sensors.

[44] Christison G.B., MacKenzie H.A. (1993). Laser photoacoustic determination of physiological glucose concentrations in human whole blood. Med. Biol. Eng. Comput..

[45] Fakhlaei R., Babadi A.A., Sun C., Ariffin N.M., Khatib A., Selamat J., Xiaobo Z. (2024). Application, challenges and future prospects of recent nondestructive techniques based on the electromagnetic spectrum in food quality and safety. Food Chem..

[46] Yadav J., Rani A., Singh V., Murari B.M. (2015). Prospects and limitations of non-invasive blood glucose monitoring using near-infrared spectroscopy. Biomed. Signal Process. Control.

[47] Burmeister J.J., Arnold M.A. (1999). Evaluation of Measurement Sites for Noninvasive Blood Glucose Sensing with Near-Infrared Transmission Spectroscopy. Clin. Chem..

[48] Olesberg J.T., Arnold M.A., Mermelstein C., Schmitz J., Wagner J. (2005). Tunable Laser Diode System for Noninvasive Blood Glucose Measurements. Appl. Spectrosc..

[49] Liakat S., Bors K.A., Huang T.-Y., Michel A.P.M., Zanghi E., Gmachl C.F. (2013). In vitro measurements of physiological glucose concentrations in biological fluids using mid-infrared light. Biomed. Opt. Express.

[50] Rosencwaig A. (1973). Photoacoustic Spectroscopy of Biological Materials. Science.

[51] MacKenzie H.A., Ashton H.S., Spiers S., Shen Y., Freeborn S.S., Hannigan J., Lindberg J., Rae P. (1999). Advances in Photoacoustic Noninvasive Glucose Testing. Clin. Chem..

[52] El-Busaidy S., Baumann B., Wolff M., Duggen L., Bruhns H. (2019). Experimental and Numerical Investigation of a Photoacoustic Resonator for Solid Samples: Towards a Non-Invasive Glucose Sensor. Sensors.

[53] Cox B., Laufer J.G., Arridge S.R., Beard P.C. (2012). Quantitative spectroscopic photoacoustic imaging: A review. J. Biomed. Opt..

[54] Rosenthal A., Ntziachristos V., Razansky D. (2011). Model-based optoacoustic inversion with arbitrary-shape detectors. Med. Phys..

[55] Nie L., Chen X. (2014). Structural and functional photoacoustic molecular tomography aided by emerging contrast agents. Chem. Soc. Rev..

[56] Baumann B., Kost B., Wolff M., Groning H. (2008). Modeling and Numerical Investigation of Photoacoustic Resonators. Modelling and Simulation.

[57] Duggen L., Lopes N., Willatzen M., Rubahn H.-G. (2011). Finite Element Simulation of Photoacoustic Pressure in a Resonant Photoacoustic Cell Using Lossy Boundary Conditions. Int. J. Thermophys..

[58] Glière A., Rouxel J., Brun M., Parvitte B., Zéninari V., Nicoletti S. (2014). Challenges in the Design and Fabrication of a Lab-on-a-Chip Photoacoustic Gas Sensor. Sensors.

[59] Baumann B., Wolff M., Kost B., Groninga H. (2007). Finite element calculation of photoacoustic signals. Appl. Opt..

[60] Pleitez M.A., Lieblein T., Bauer A., Hertzberg O., von Lilienfeld-Toal H., Mäntele W. (2013). Windowless ultrasound photoacoustic cell for in vivo mid-IR spectroscopy of human epidermis: Low interference by changes of air pressure, temperature, and humidity caused by skin contact opens the possibility for a non-invasive monitoring of glucose in the interstitial fluid. Rev. Sci. Instrum..

[61] Sim J.Y., Ahn C.-G., Jeong E.-J., Kim B.K. (2018). In vivo Microscopic Photoacoustic Spectroscopy for Non-Invasive Glucose Monitoring Invulnerable to Skin Secretion Products. Sci. Rep..

[62] El-Busaidy S., Baumann B., Wolff M., Duggen L. (2021). Shape optimization of an open photoacoustic resonator. Appl. Sci..

[63] Camou S., Haga T., Tajima T., Tamechika E. (2012). Detection of aqueous glucose based on a cavity size- and optical-wavelength-independent continuous-wave photoacoustic technique. Anal. Chem..

[64] Sensors Council 2013 IEEE sensors. Proceedings of the 12th IEEE Sensors Conference.

[65] Wadamori N. (2015). Behavior of long-period measurements using a small-sized photoacoustic cell for aqueous glucose monitoring. Proceedings of the 2015 37th Annual International Conference of the IEEE Engineering in Medicine and Biology Society (EMBC).

[66] Sim J.Y., Ahn C., Jeong E., Kim B. Photoacoustic spectroscopy that uses a resonant characteristic of a microphone for in vitro measurements of glucose concentration. Proceedings of the Engineering in Medicine and Biology Society IEEE.

[67] Zhao S., Tao W., He Q., Zhao H., Yang H. (2017). Glucose solution determination based on liquid photoacoustic resonance. Appl. Opt..

[68] El-Busaidy S.A.S., Baumann B., Wolff M., Duggen L. (2020). Modelling of open photoacoustic resonators. Photoacoustics.

[69] Yang L., Chen C., Zhang Z., Wei X. (2022). Glucose Determination by a Single 1535 nm Pulsed Photoacoustic Technique: A Multiple Calibration for the External Factors. J. Healthc. Eng..

[70] Aloraynan A., Rassel S., Kaysir M.R., Ban D. (2023). Dual quantum cascade lasers for noninvasive glucose detection using photoacoustic spectroscopy. Sci. Rep..

[71] ISHIHARA Y., WADAMORI N. (2008). A Study on Enhancement of Sensitivity of a PhotoAcoustic Detector Cell for Non-invasive Measurements Based on Finite Element Method Analysis(Symposium on Biomedical Engineering 2007). Trans. Jpn. Soc. Med. Biol. Eng. BME.

[72] Tachibana K., Okada K., Kobayashi R., Ishihara Y. Development of a high-sensitivity and portable cell using Helmholtz resonance for noninvasive blood glucose-level measurement based on photoacoustic spectroscopy. Proceedings of the 2016 38th Annual International Conference of the IEEE Engineering in Medicine and Biology Society (EMBC).

[73] Takamoto R., Namba R., Matsuoka M., Sawada T. (1992). Human in vivo percutaneous absorptiometry using the laser-photoacoustic method. Anal. Chem..

[74] Takamoto R., Yamamoto S., Namba R., Takamatsu T., Matsuoka M., Sawada T. (1994). In vivo Percutaneous Absorptiometry by a Laser Photoacoustic Method Using a Novel Open-Ended Cell Combined with Light Guide. Anal. Chem..

